# Severely Asthmatic Horses Residing in a Mediterranean Climate Shed a Significantly Lower Number of Parasite Eggs Compared to Healthy Farm Mates

**DOI:** 10.3390/ani13182928

**Published:** 2023-09-15

**Authors:** Joana Simões, José Paulo Sales Luís, Luís Madeira de Carvalho, Paula Tilley

**Affiliations:** 1 Equine Health and Welfare Academic Division, Faculty of Veterinary Medicine, Lusófona University, Campo Grande 376, 1749-024 Lisbon, Portugal; 2 CIISA—Centre for Interdisciplinary Research in Animal Health, Faculty of Veterinary Medicine, University of Lisbon, 1300-477 Lisbon, Portugal; jpluis@fmv.ulisboa.pt (J.P.S.L.); madeiradecarvalho@fmv.ulisboa.pt (L.M.d.C.); ptilley@fmv.ulisboa.pt (P.T.); 3 Associate Laboratory for Animal and Veterinary Sciences (AL4Animals), Faculty of Veterinary Medicine, University of Lisbon, 1300-477 Lisbon, Portugal

**Keywords:** severe equine asthma, nematode, horse strongyles, cyathosmin, Mediterranean climate

## Abstract

**Simple Summary:**

Severe equine asthma is an allergic disease and commonly affects adult horses, resulting in increased respiratory effort, nasal discharge and cough. Human patients affected by allergic diseases tend to present a lower gastrointestinal parasitic burden, when compared to healthy individuals. The present study intended to ascertain if this tendency would also be observed in a group of non-related severely asthmatic horses living in a Mediterranean climate. Thus, the number of gastrointestinal parasites shed by severely asthmatic and non-asthmatic horses from the same farms subjected to a similar management and deworming program were compared. The horses diagnosed with severe equine asthma shed a lower number of parasitic eggs and infective larvae in comparison to their healthy mates. This being a brief report, there is still the need to ascertain if this occurs in a larger population of horses; in addition, further research is necessary to understand the mechanisms behind this process.

**Abstract:**

The relationship between helminth infection and allergic diseases has long intrigued the scientific community. This interaction was previously studied in a horse family with high incidence of severe equine asthma and in non-related severely asthmatic horses from equine hospital referrals in Switzerland. Our aim was to determine if this interaction would also be observed in a group of non-related client-owned severely asthmatic horses living in a Mediterranean climate and recruited through a first-opinion veterinarian group. Fecal samples from severe equine asthma-affected and healthy horses living in the same farms and subjected to identical environmental and deworming management were evaluated qualitatively and quantitatively. Strongyle-type eggs and *Cyathostomum sensu latum* larvae were the most abundant parasites in the studied population of horses; no significant differences between the groups were observed regarding the types of egg and infective larvae. However, we observed significant differences in the number of eggs and infective larvae per gram of feces shed, as this number was significantly lower in the SEA group than in the healthy horses. This may indicate that severely asthmatic horses have an intrinsic resistance to gastrointestinal helminths. Further studies in a larger population of horses are required to ascertain the immunological mechanisms responsible for these findings.

## 1. Introduction

In humans, the prevalence of allergic diseases has been increasing at an astounding rate in Western industrialized countries [1,2,3]. However, due to the lack of large-scale multi-center epidemiological studies, this trend has yet to be confirmed in equine medicine [4]. Severe equine asthma (SEA) is a highly prevalent respiratory disease affecting horses mostly in the Northern hemisphere and is thought to have an allergic component [5,6,7]. Severely asthmatic horses develop lower airway inflammation and obstruction associated with coughing, increased respiratory effort at rest, decreased clearance and increased mucus production, as well as exercise intolerance in response to the exposure to respirable dust particles [8,9,10].

In the last two decades, an association between allergic diseases and intestinal parasite infection has been extensively reported in humans. Moreover, although explanatory theories differ in proposed causality, allergic individuals seem to present lower parasitic counts than their healthy counterparts [11,12,13]. It has been proposed that protection against intestinal nematodes may have been an evolutionary advantage, which due to a common genetic basis, increased the risk for allergic diseases [13,14]. Alternatively the ‘hygiene hypothesis’ suggests that the disappearance of chronic infectious diseases resulted in the loss of cellular and humoral immunoregulatory pathways, contributing to the development of allergic diseases [2,15]. This hypothesis suggests that a lack of exposure to bacterial antigens, that would otherwise lead to a more T-helper1-dominated response, combined with low levels of parasite-specific IgE, will reduce the threshold at which the IgE response to environmental antigens can sensitize mast cells [16,17,18]. In fact, with the discovery of the gut–lung axis and the two-way relationship between these compartments, it has become increasingly accepted that the gut parasitome may in fact have an immunomodulatory effect by modifying the bacterial composition of the intestine and regulating the Th1/Th2/Treg response [19].

Conflicting results have been reported on the relationship between the gastrointestinal parasitic burden and severe equine asthma, but these studies focused mainly on two horse families with a high prevalence of SEA and on non-related SEA-affected horses from equine hospital referrals in Switzerland [13,20]. 

We hypothesized that an inverse relationship may occur in non-related, client-owned, severely asthmatic horses living in a Mediterranean climate and recruited through a first-opinion veterinarian group. To test this hypothesis, we compared the helminth composition and burden of a group of SEA-affected horses with those of healthy mates from the same farms.

## 2. Materials and Methods

### 2.1. Horses

The present study was approved by the Ethics and Welfare Committee of the Faculty of Veterinary Medicine of the University of Lisbon, and individual informed owner consent was obtained for each horse, previous to its enrollment in the study.

The sample size was estimated in order to identify an effect size of 40%, using an alpha of 0.05 and a statistical power of 80%. The drop-out rate was assumed to be 0%, since after enrollment the animals would be evaluated only once. The sample size estimation also involved the examination of previously published studies with similar outcomes, such as the ones published by Neuhaus et al. [13] and by Bründler et al. [20], which reported an effect size between 10 and 21%. However, we opted for a larger effect size, because our cases would comprise non-related horses from different areas of Portugal recruited via an equine veterinarian group in a social media app (WhatsApp Inc. (Facebook, Inc., Menlo Park, CA, USA). https://whatsapp.com), involving first-opinion veterinarians in ambulatory practice. The horse owners were then asked to take the horses to the hospital for the staging of asthma. 

Forty (n = 40) non-related client-owned horses living in Portugal, with ages comprised between 9 and 16 years (mean age 13 ± 2 years) were enrolled in this study. The large majority of the animals were males (30 geldings and 2 stallions), and only 8 were mares. The horses were divided in two groups of 20 animals each, according to their respiratory disease status—a SEA group and a control group—and each severely asthmatic animal was matched with a healthy control from the same farm of a similar age and, when possible, of the same gender. The latter was only possible for 16 of the 20 SEA–control pairs.

All animals from both groups had been exposed to hay in the six months before the study and were vaccinated for influenza. The owners reported that severely asthmatic horses had a history of cough, nasal discharge and/or respiratory effort at rest, whilst the animals enrolled in the control group had no similar complaints.

Furthermore, the inclusion of each animal in either the SEA or the control group was determined by the results of the SEA staging method developed by Tilley et al. [9]. This method includes physical, radiographic, endoscopic and cytological examination for the diagnosis and staging of SEA. Firstly, cough frequency and severity of nasal flare and of abdominal lift were assessed to determine the clinical score. Thoracic radiography was then performed to assess the trachea and lungs and to determine the X-ray score. This was followed by respiratory endoscopy to evaluate the characteristics of the tracheal mucus, which were used to determine the endoscopic score. Finally, a cytological analysis of the bronchoalveolar lavage fluid (BALF) was performed, and the percentage of neutrophils was used to determine the BALF score. The sum of each parameter’s scores (clinical score + X-ray score + endoscopic score + BALF score) was then used to determine the SEA stage, with severely asthmatic animals presenting a stage score ≥1, and healthy horses had a stage score = 0.

Horses with a history of clinical signs compatible with equine asthma but with a SEA stage of <1 were excluded from the study. Additionally, if the ancillary tests or the BALF cytology presented findings compatible with other clinical entities or disease complications, such as bacterial contamination, the horses were excluded from the study.

Each SEA–control pair was subjected to the same deworming and vaccination protocol and was exposed to the same environmental management. 

All horses were usually dewormed with 200 µg/kg bw of ivermectin (n = 40), and the treatment frequency was either yearly (n = 18) or twice a year (n = 22). Two to three months prior to enrollment in this study, all animals received an anthelmintic treatment. 

Each pair (SEA–control horse) was placed in the same housing conditions, received a similar diet and had access to the same spaces. 

### 2.2. Faecal Sample Collection and Analysis

Fecal samples were obtained directly from the rectum or from freshly void feces and were examined on the same day. Sample collection was performed simultaneously for each SEA–control pair.

A quantitative and qualitative analysis of fecal eggs and larvae was performed for each sample.

#### 2.2.1. Egg Count and Identification

Egg count was determined with the McMaster method, and identification was performed using the flotation and sedimentation methods.

The number of strongyle eggs per gram of feces (EPG) was assessed with the McMaster technique. Two grams of the homogenized sample was diluted in 28 mL of a 25% sugar solution with a 1.2–1.25 density. After filtration, the sample was placed in a McMaster chamber, and the number of eggs was counted using an optical microscope with a 10× objective. The number of eggs was multiplied by 50 to obtain the total EPG [21,22]. 

With the remaining fecal suspension, a test tube was filled, and a coverslip was placed on top. After 15 min, the eggs attached to the coverslip were identified through microscopic observation [21,22].

The excess supernatant liquid from the previous technique was discarded, and the sediment was stained with methylene blue, homogenized and observed under a microscope for egg identification [21,22].

#### 2.2.2. Infective Larvae Count (L3) and Identification

Approximately 47 g (±10 g) of each homogenized fecal sample was deposited in a cup, and a small hole was made in the middle of each sample. The cups were then covered with pierced aluminum foil and stored in an incubator at 26–28 °C along with a container with 500 mL of water to provide moisture. After 14 days, the samples were removed from the incubator, and after discarding the aluminum foil, the cups were filled with water and covered with a Petri dish. The cups were then inverted, in order to completely fill the Petri dish with water and were left undisturbed for 24 h. The liquid was then collected into 10 mL centrifuge tubes, and the obtained samples were centrifuged at 377 g for 5 min. An aliquot of 100 µL was observed under the microscope in order to count and identify the strongyle infective larvae stages (L3) [22]. The identification of horse strongyle L3 to the genus/species level was performed according to Madeira de Carvalho et al. [23].

The number of infective larvae per gram (LPG) of feces was calculated according to Equation (1):

LPG = (N × F)/W(1)

LPG—Larvae per gram of feces; N—Number of larvae per 100 µL; F—Dilution factor to reach the total volume of water (100); W—Weight of the sample in grams.

### 2.3. Statistical Analysis

The results were analyzed using IBM SPSS Statistics software (version 25.0). Data normality was assessed using the Shapiro–Wilk test, and because the data did not present a normal distribution, the differences between the two groups, namely, egg and infective larvae (L3) counts, were analyzed using the Mann–Whitney U test. The differences were considered significant if *p* < 0.05. The results are presented in the form of medians and interquartile ranges (IQR).

## 3. Results

### 3.1. Egg Count and Identification

**Figure 1:**
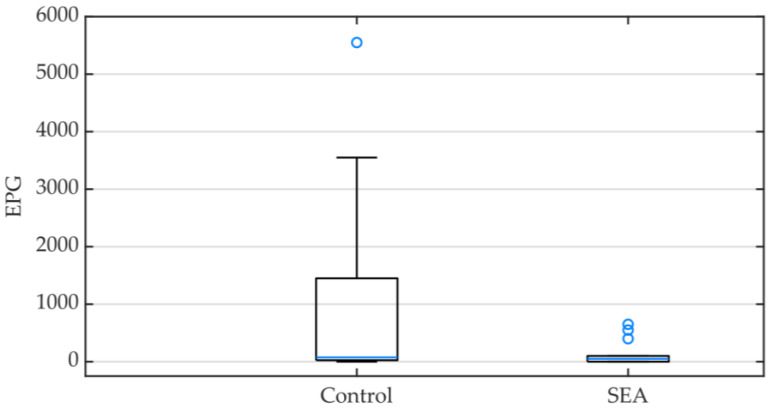
Box plot representing the egg count per gram (EPG) of feces in the control and the severe equine asthma (SEA) groups. Minimum, median (blue line) and maximum values as well as interquartile ranges are displayed.

A total of 24 animals (60%) presented a positive egg count; the EPG value of each animal is available in the Supplementary Materials, Table S1. The median EPG was higher in the control group (75, IQR 37.5–1300) than in the SEA group (50, IQR 0–100) and significantly different between the two groups (*p* = 0.043) (Figure 1).

Using the flotation method, nematode eggs were observed in 24 animals (60%), and 11 animals (28.9%) also presented eggs in the sediment. The majority of the eggs observed were strongyle-type (95%), and a small number corresponded to *Strongyloides westeri* eggs (5%). The latter were observed in the fecal sample of one animal enrolled in the control group, whilst strongyle-type eggs were present in all animals which were positive for nematode eggs.

### 3.2. Infective Larvae (L3) Count and Identification

**Figure 2:**
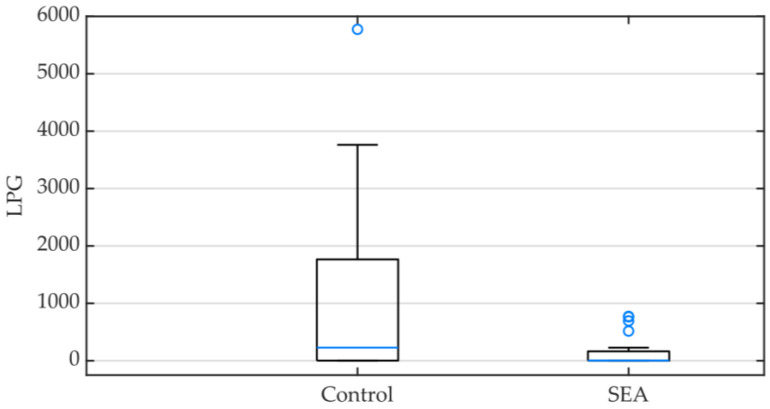
Box plot representing infective larvae count per gram (LPG) of feces in the control and the severe equine asthma (SEA) groups. Minimum, median (blue line) and maximum values as well as interquartile ranges are displayed.

As expected, based on the EPG results, 24 animals (60%) had a positive L3 count; the information pertaining to each animals’ LPG is available in the Supplementary Materials, Table S1. When comparing the SEA and the control groups, significant statistical differences were found (*p* = 0.040), and the control group LPG median was considerably higher (229, IQR 3–1766) than that of the SEA group (3.5, IQR 0–131.25) (Figure 2).

Infective larval stages (L3) were observed in 24 animals (60%) in the studied population, and regarding their abundance, 0.11% of all L3 larvae observed were identified as *Triodontophorus* spp., 0.23% as *Poteriostomum* spp., 27.93% as *Strongyloides westeri*, and 71.73% as *Cyathostomum sensu latum*. *Cyathostomum sensu latum* larvae were highly prevalent in both groups of horses. However, in the control group, *Triodontophorus* spp. and *Poteriostomum* spp. larvae were identified in one of the fecal samples, and *Strongyloides westeri* in another. These species were not observed in the corresponding SEA horse feces (Figure 2).

## 4. Discussion

Allergic diseases are considered polygenetic disorders depending not only on genetics and exposure to allergens, but also on a dysregulation of the immune response; these three factors have been implicated in the pathogenesis of SEA [24,25,26,27,28]. However, the exact immune mechanisms responsible for this disease remain unknown, and conflicting reports of the immunological pathways involved in SEA have led to the hypothesis that multiple endotypes (molecular and genetic mechanisms) may be responsible for the same phenotype [5,27].

In horses, intestinal parasites have been recognized as a cause of clinical disease since the Roman Empire [29] with the emergence of the *equorum medicus*, veterinarian assistant of the mounted Roman army, and the writing of the book Hippiatria by Apsyrtus. Nowadays, helminths, such as strongyles, continue to be a cause of acute and chronic gastrointestinal disease with varying degrees of severity [30,31]. However, in humans and, most recently, in horses, an association between allergic diseases and intestinal parasite infection has been extensively documented [1,2,11,12,13,20].

Amongst the most popular explanations for this association proposed by the scientific community, there are hygiene and genetic hypothesis. The first states that the enforcement of hygiene habits reduced the prevalence of chronic infectious diseases, such as intestinal parasitism [2,15,32]. Nowadays, this hypothesis has gained new momentum, supported by different studies which report the role of the microbiome as a modulator of the hosts’ immune response [33]. Acute helminth infection is associated with a local innate response and an adaptive Th2-type response, but in chronic stages, these parasites also have the ability to suppress the immune system by increasing the levels of interleukin (IL)-10 and regulatory T cells, which could explain the inverse relationship of these infections with allergies, such as asthma and atopy [19,34,35]. Furthermore, it has been proposed that prenatal infection by helminths may decrease the risk of allergic diseases [36,37]. In a mouse model, pregnancy during the chronic stage of schistosome infection prevented the development of asthma [37].

On the other hand, the genetic hypothesis theorizes that the genetic makeup of allergic individuals grants them the advantage of being more resistant to parasitic infections, while at the same time conferring an increased vulnerability to environmental allergens. Thus, it is believed that both traits have a common genetic basis [13,14].

The latter hypothesis was supported by the findings reported for two different Swiss Warmbloods families with a high prevalence of SEA, revealing that one family possessed increased resistance to helminth parasites [13], whilst the other did not [20]. In the family where an inverse relationship between parasites and SEA was identified, disease inheritance was described as being autosomal recessive and associated with *IL4Rα* and its neighboring regions in the ECA13 chromosome [25,38]. The *IL4Rα* gene has also been associated with asthma in humans [39,40]. However, in the second family, disease inheritance was autosomal dominant and linked to the ECA15 region [26]. These genetic differences may affect horses’ susceptibility to helminth infection.

Our findings, in a group of non-related SEA-affected horses living in a Mediterranean climate and recruited through a first-opinion veterinarian group, are similar to those reported for one of the Swiss Warmblood families, indicating a similar tendency in a group of non-related severely asthmatic horses living in Portugal. A similar finding was reported for a group of non-related SEA-affected horses from hospital referrals living in Switzerland [20], which could lead to the hypothesis that genetic mechanisms associated with the ECA13 and *IL4Rα* neighboring regions are common in European horses, and, like in humans, polymorphic differences in the *IL4Rα* gene may play a role in the development of SEA [39,40]. 

Strongyle-type eggs and *Cyathostomum sensu latum* infective larvae were the most abundant species identified in the studied population. These nematodes have become highly prevalent in the modern era and are a frequent cause of gastrointestinal disease in horses [30,41,42]. They have also been commonly reported in equids living in Portugal [22,23,30,43], explaining their abundance in the studied population. However, some nematode species identified in the control animals were not present in the SEA group, namely, *Strongyloides westeri*, *Triodontophorus* spp. and *Poteriostomum* spp. It could be that the severely asthmatic horses had an increased resistance to these infectious species, although conclusions cannot be extrapolated from this small population, and further research is required to assess this hypothesis. To the authors knowledge, very little information is available about this subject since most studies report quantitative but not qualitative differences between the gastrointestinal parasitism of SEA-affected and healthy horses [13,20].

Because SEA is a disease that is characteristic of older animals [5,44], the horses enrolled in this study were all adults, and each SEA–control pair was approximately the same age (mean age difference, 2 years ± 2.6 years). Additionally, age is considered a risk factor for parasite infection, with young animals presenting an increased shedding of helminth eggs [45,46]. A gender-based pairing between the severely asthmatic horses and their farm mates was not always possible, since some farms only possessed adult animals of a specific gender. Nonetheless, gender is not a predisposing factor for the development of SEA [5,44], and recent studies reported no significant differences in the excretion of helminth eggs between genders for animals of the same age, kept under a similar management program [45,46,47,48]. For this reason, we prioritized pairing the animals according to their age rather than their gender, with 16 out of the 20 SEA–control pairs still having the same gender.

All the enrolled animals were non-related and client-owned, and their inclusion in the study required a thorough diagnostic process to assess their respiratory status, including the attainment of their clinical history and the disease staging according to Tilley et al. [9]. However, we cannot rule out the hypothesis that some of the control animals may have been misdiagnosed as healthy and were in fact cases of mild asthma, though their owners did not report any signs of the disease, such as loss of performance, since we did not rely on lung function tests to differentiate the two groups. Nonetheless, the control horses had a BALF cytology compatible with that of a healthy horse and no history of clinical respiratory disease in the last year, even though they were equally exposed to hay in the 6 months prior to the start of the study.

Additionally, because of the small size of the sample used in this brief report, a careful interpretation of the obtained results is recommended, since the data did not have a normal distribution and could present some bias. Although we predicted in our initial sample calculation that forty animals would allow us to assess the potential differences between the groups, we still have to consider the high number of horses with egg and infective larvae counts equal to zero. Thus, further studies with a larger number of client-owned non-related asthmatic horses are required to assess if the inverse relationship between SEA and gastrointestinal parasites is also present in other affected animals.

Nonetheless, in the present study, we observed significant differences in parasite burden between the two groups and between the two animals in most of the SEA–control pairs. The control horses shed on average, nine times more eggs (median, 1.5 times more eggs) and ten times more infective larvae (median, 65 times more larvae) than the severely asthmatic horses. 

With this brief report, we hope to alert researchers for the need to further investigate the potential mechanisms underlying the differences in parasite burden observed between the two groups examined and better understand the role of the equine parasitome in disease and health. For this purpose, large-scale multi-center studies are essential to evaluate not only the disease epidemiology, but also the molecular and genetic mechanisms associated with SEA and ascertain the true nature of the relationship between this respiratory disease and the apparent resistance of affected individuals to gastrointestinal helminths.

## 5. Conclusions

The population of severely asthmatic horses included in this study, living in a Mediterranean climate, shed a significantly lower number of parasite eggs (lower mean EPG), when compared to healthy mates with similar deworming and environmental management.

Regardless, the disease status seemed to have no impact on the infective nematode species, since strongyle-type eggs and cyathostomin larvae were highly abundant and prevalent in both groups. This reflects the reported trends for helminth infections in equids in Portugal [22,23,43].

Although several hypotheses on the link between asthma and lower parasitic burdens have been proposed, whether there is a specific immunological mechanism behind this finding still remains to be understood. Further understanding of the role of helminths and other gastrointestinal parasites in the modulation of the immune response can help ascertain if these organisms hinder the development of SEA or if asthmatic animals are in fact more resistant to this type of infection.

## Data Availability

The data presented in this study are available in Supplementary Materials.

## Supplementary Materials

The following supporting information can be downloaded at: https://www.mdpi.com/article/10.3390/ani13182928/s1, Table S1: Total number of eggs per gram of feces and total number of larvae per gram of feces in each pair of SEA–control mates and percentual difference between mates.
