# Supplementary material for: Severely Asthmatic Horses Residing in a Mediterranean Climate Shed a Significantly Lower Number of Parasite Eggs Compared to Healthy Farm Mates
Source: Animals (Basel). 2023 Sep 15;13(18):2928. doi: 10.3390/ani13182928 (PMC10525552; doi:10.3390/ani13182928)
Supplement: Supplementary file 1 [file animals-13-02928-s001.zip › animals-2562265-supplementary.pdf]

Table S1. Total number of eggs per gram of feces and total number of larvae per gram of feces counted each pair of SEA-Control mates and percentual difference between mates.

	EPG			LPG		
Pair	SEA group	Control group	Percentual difference	SEA group	Control group	Percentual difference
1	50	3050	30	10	2860	28.5
2	0	50	0.5	4	14	0.1
3	0	0	0	0	0	0
4	550	1150	6	517	807	2.9
5	100	1800	17	99	1766	16.67
6	50	50	0	0	0	0
7	100	1750	16.5	228	3762	35.34
8	0	50	0.5	0	4	0.04
9	0	5550	55.5	0	430	4.3
10	50	0	-0.5	19	0	-0.19
11	50	1000	9.5	694	1232	5.38
12	0	0	0	0	0	0
13	0	0	0	0	4	0.04
14	400	100	-3	765	28	-7.37
15	0	50	0.5	0	1766	17.66
16	100	1050	9.5	7	1926	19.19
17	650	3550	29	773	5774	50.01
18	0	0	0	0	0	0
19	50	600	5.5	3	756	7.53
20	0	50	0.5	0	5	0.05
